# A systematic evaluation of the error detection abilities of a new diode transmission detector

**DOI:** 10.1002/acm2.12691

**Published:** 2019-08-05

**Authors:** Vikren Sarkar, Adam Paxton, Jeremy Kunz, Martin Szegedi, Geoff Nelson, Prema Rassiah‐Szegedi, Hui Zhao, Y. Jessica Huang, Frances Su, Bill J. Salter

**Affiliations:** ^1^ University of Utah Salt Lake City UT USA

**Keywords:** error detection, IMRT, quality assurance, transmission detector

## Abstract

Transmission detectors meant to measure every beam delivered on a linear accelerator are now becoming available for monitoring the quality of the dose distribution delivered to the patient daily. The purpose of this work is to present results from a systematic evaluation of the error detection capabilities of one such detector, the Delta^4^ Discover. Existing patient treatment plans were modified through in‐house‐developed software to mimic various delivery errors that have been observed in the past. Errors included shifts in multileaf collimator leaf positions, changing the beam energy from what was planned, and a simulation of what would happen if the secondary collimator jaws did not track with the leaves as they moved. The study was done for simple 3D plans, static gantry intensity modulated radiation therapy plans as well as dynamic arc and volumetric modulated arc therapy (VMAT) plans. Baseline plans were delivered with both the Discover device and the Delta^4^ Phantom+ to establish baseline gamma pass rates. Modified plans were then delivered using the Discover only and the predicted change in gamma pass rate, as well as the detected leaf positions were evaluated. Leaf deviations as small as 0.5 mm for a static three‐dimensional field were detected, with this detection limit growing to 1 mm with more complex delivery modalities such as VMAT. The gamma pass rates dropped noticeably once the intentional leaf error introduced was greater than the distance‐to‐agreement criterion. The unit also demonstrated the desired drop in gamma pass rates of at least 20% when jaw tracking was intentionally disabled and when an incorrect energy was used for the delivery. With its ability to find errors intentionally introduced into delivered plans, the Discover shows promise of being a valuable, independent error detection tool that should serve to detect delivery errors that can occur during radiotherapy treatment.

## 1 INTRODUCTION

In recent years, the complexity of radiotherapy treatment has increased considerably. Not only are modulated delivery techniques producing extremely complex dose distributions that are highly conformal to the target, but there has also been a simultaneous increase in dependence on complex imaging modalities to ensure the required accuracy of alignment to the target.^1^, ^2^, ^3^, ^4^, ^5^ Studies have shown that such increases in complexity can result in higher risks of errors occurring.^6^, ^7^, ^8^

A relatively recent series of high profile newspaper articles highlighted some of the worst of these errors.^9^ This has led our field towards a heightened awareness of and attention towards strategies of mitigation for such mistakes. Currently, significant effort is being focused on benefitting from lessons learned in other industries to better understand and eliminate errors at their source.^10^, ^11^ Efforts include application of principles of process engineering, including failure mode effects analysis^12^, ^13^ and fault tree analysis,^14^, ^15^ to identify the highest impact fault modes, along with efforts to organize incident learning systems to collect, study, and learn from errors.^16^, ^17^

Some approaches lead to detailed checklists, but research has shown that a risk of “checklist fatigue” occurs when such lists grow to be too long, thus, defeating their primary purpose.^18^ Other groups have chosen to focus their efforts on the development of automated approaches, using software and/or hardware, to automatically identify potentially dangerous discrepancies.^19^, ^20^, ^21^, ^22^, ^23^, ^24^, ^25^, ^26^, ^27^, ^28^ Some software approaches evaluate plan quality to ensure that planners did not inadvertently fail to achieve important quality metrics.^29^ Another approach utilizes radio‐frequency identification technology and associated software to ensure that required patient setup devices are both present and in the correct location and orientation.^30^ Another category of automated equipment/software strives to measure the daily delivered dose distribution either by evaluating the entrance^21^, ^22^, ^23^, ^25^, ^26^, ^27^, ^31^, ^32^ or exit fluence.^33^, ^34^, ^35^

**Figure 1:**
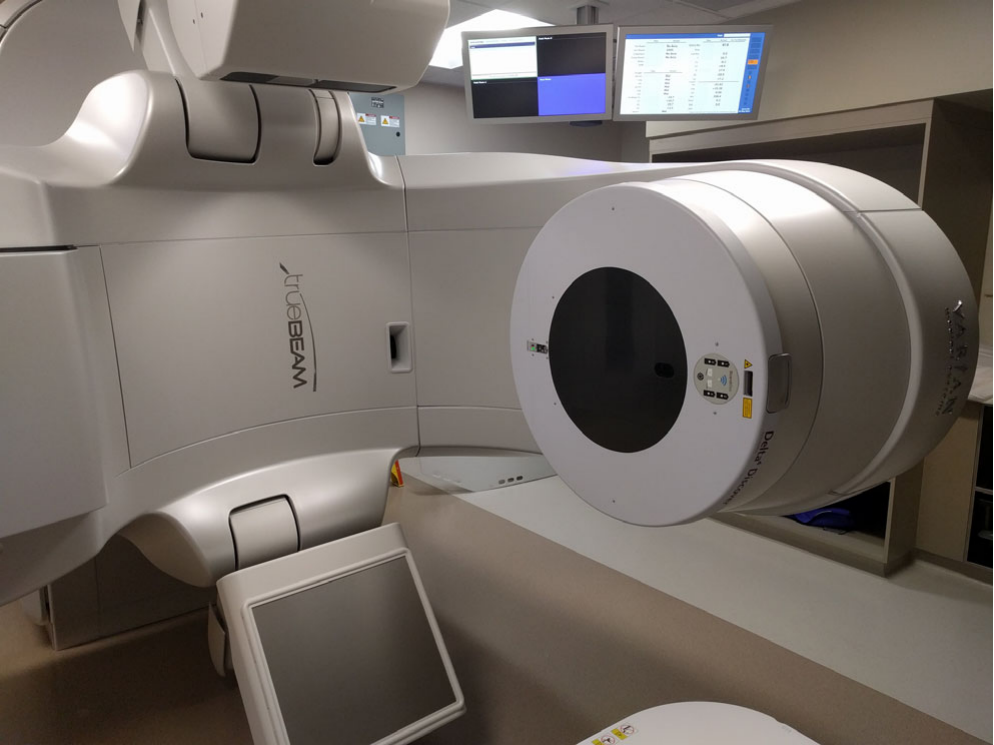
Discover device mounted on a linear accelerator head.

One of the disadvantages of using exit dosimetry is that the exit dose depends on both machine performance and patient geometry. While devices that measure entrance fluence cannot predict changes in dose delivered to the patient stemming from changes in patient geometry, detailed knowledge of the fluence delivered by the linear accelerator can be used to calculate the dose to the patient using the patient's geometry. The Discover device^23^ is a hardware device that is designed to measure the daily delivered entrance fluence and is shown in Fig. 1 mounted on a linear accelerator. The detector is made up of 4040 diode detectors separated by 2.5 mm along the multileaf collimator (MLC) motion direction. The diodes are separated by 5 mm perpendicular to the direction of motion of the MLC leaves. The area covered is 19.5 × 25 cm when projected to isocenter level. The detector is attached to the head of the linac, allowing the treatment beam to pass through with relatively little attenuation (~1%). The associated software compares the fluence pattern, as it is delivered to the patient, to the intended distribution and, as such, serves as a near real‐time, automated error detection system. The clinical value of such a system is inherently tied to its sensitivity, as well as any limitations in its ability to detect errors. This work systematically evaluated the ability of the Discover system to detect multiple delivery errors that were intentionally introduced into treatment plans, and we report on the sensitivity with which the device detected such errors and any limitations in error detection ability.

## 2 MATERIALS AND METHODS

As a fluence measurement device, the Discover (ScandiDos, Uppsala, Sweden) is meant to be used during every treatment to monitor the fidelity of the dose distribution delivered. There are two different ways that the Discover can be used: (a) by itself or (b) in conjunction with a phantom during a separate quality assurance (QA) session. When used by itself, the use of the Discover is referred to as “Express Measure” and the device can provide information on the location of the MLC leaves, as well as the gantry and collimator. However, while the device is measuring fluence, there is presently not a way to convert these measurements to a dose distribution. This is because the signal level measured varies with the full geometric characteristics of the aperture used which, in effect, determines the scatter conditions. Furthermore, the proximity of the detector to the treatment head causes the detector to see contaminant electrons that are never seen by a phantom traditionally used in dose validation. In order to convert the fluence to a dose, the device can be used in “Synthesis Mode,” in conjunction with the Delta4 Phantom+ (D4+) that directly measures dose. This device is made up of 1069 diode detectors arranged along the coronal and sagittal planes. Detectors are located 5 mm apart in the central area and 1 cm apart otherwise and cover a maximum field size of 20 × 38 cm^2^.

Since the signal level from both devices can be synchronized, and the D4+ measures dose for each control point, the Scandidos software (version August 2018, Scandidos AB) can create a link between the signal levels from the Discover and the dose measurements in the D4+ for each control point. Following this initial synchronization measurement (in Synthesis Mode) future Discover measurements can be performed during treatment, without the D4+ device present, and fluence measurements can be “synthesized” into a dose distribution in the D4+.

For all measurements, the Discover was used in combination with a TrueBeam linear accelerator equipped with a standard millennium 120 multileaf collimator (Varian Medical Systems, Palo Alto, CA, USA). Baseline treatment plans were created in the Eclipse treatment planning system (version 15.5, Varian Medical Systems). The resulting plan and field dose information was exported in DICOM format and imported into the Delta4 software for comparison with measurements. Baseline plans were delivered with both the Discover and D4+ so that Synthesis Mode could be used for subsequent measurements. Each baseline plan was then modified using in‐house software to systematically introduce known shifts into the MLC leaf positions or gantry/collimator angles, to simulate delivery errors. These modified plans were then delivered through the Discover device to evaluate the fidelity of delivered plan quality so that the sensitivity of the device at detecting the errors could be established.

### 2.1 Single static field

**Figure 2:**
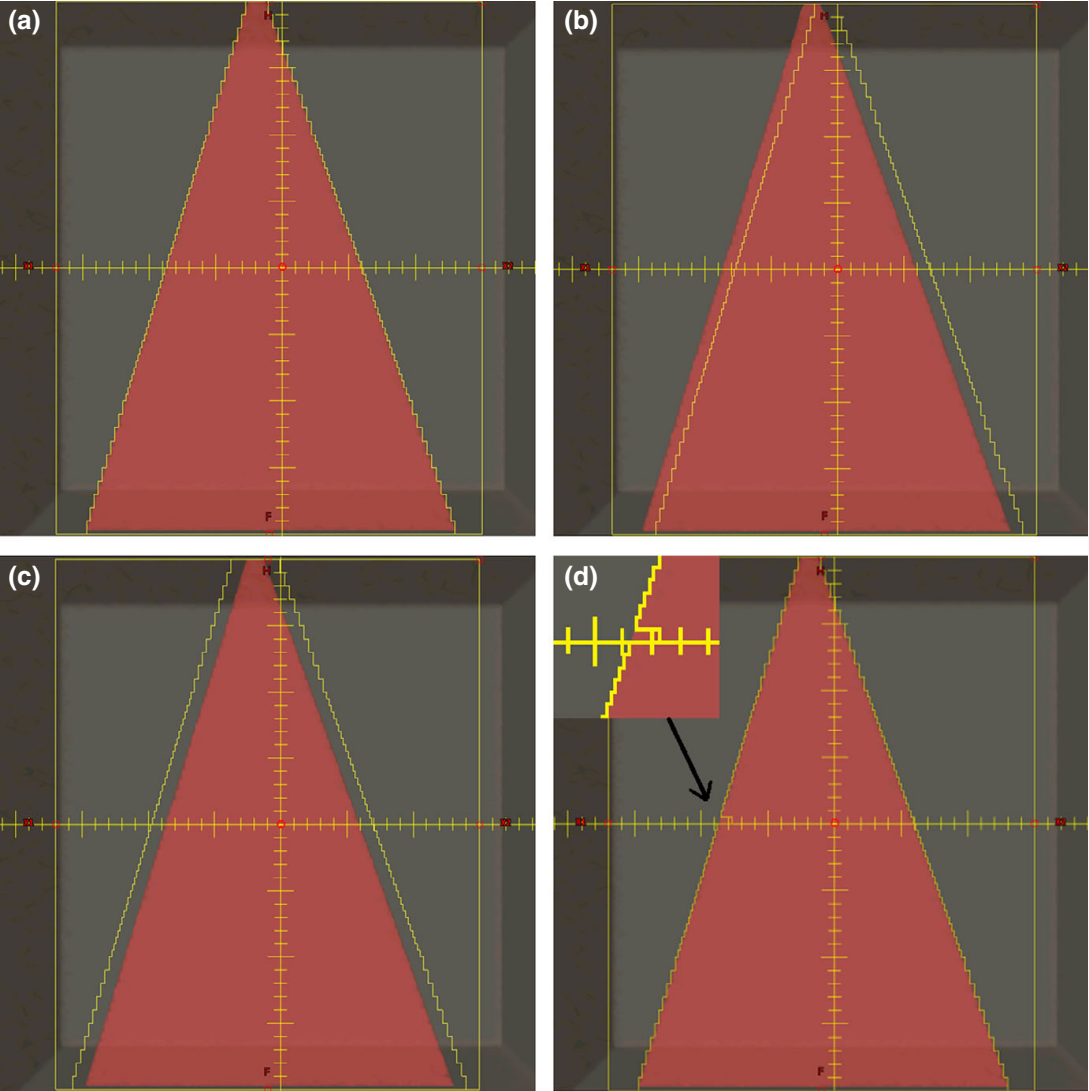
Depiction of leaf position errors introduced. (a) Baseline plan, (b) shifted leaves, (c), field opened, and (d) single central leaf moved (highlighted in the inset).

As the simplest case to investigate, a single static field was created as a base plan. In the coronal plane the field represented a triangular shape as depicted in Fig. 2(a). The dose distribution from delivering 100 MUs with the 6 MV beam was calculated on the D4+ phantom dataset. The plan file was then modified to change the MLC leaf positions by 0.25, 0.5, 1, 2, and 5 mm. Leaves were shifted using three different strategies: (a) All leaves were shifted in the same direction, (b) the field was “opened” with leaves on opposing banks shifted in opposite directions, and (c) a single central leaf was shifted. These three strategies are depicted in Figs. 2(b) to 2(d). As additional strategies, the baseline plan was modified to: move the x jaws out by 1 cm, and also to use three different photon energies instead of the original 6 MV.

#### 2.1.1 Single‐field intensity‐modulated radiation therapy (IMRT) — dynamic delivery

To slightly increase plan complexity, a single IMRT field from a clinical plan was randomly chosen. The original clinical field was planned using the 6 MV beam and utilized dynamic MLC leaf motion (dMLC) with 130 control points using the jaw‐tracking feature of Eclipse. The originally planned field was then modified by shifting all MLC leaves by 0.25, 0.5, 1, 2, and 5 mm for each segment, analogous to what was depicted in Fig. 2(b). Other errors simulated from the baseline plan included changing the beam energy and disabling jaw tracking for the field.

#### 2.1.2 Single‐field intensity‐modulated radiation therapy (IMRT) — step‐and‐shoot (SS)

The segments for the same field as in Section 2.1.1 was recalculated for delivery using step‐and‐shoot mode with jaw tracking. The resulting plan used 18 control points. This new baseline plan was also modified by shifting all leaves by 0.25, 0.5, 1, 2, and 5 mm and by disabling jaw tracking. Gamma pass rates (2%, 2 mm) were used to compare the measurements, with the pass rate from the baseline plan delivered to the phantom as the baseline value.

All modified SS‐IMRT plans were delivered twice — once using the Discover alone and once with the D4+ alone. This allowed for a direct comparison of the Synthesis‐predicted change in gamma pass rate due to introduced errors to that from the actual dose distribution measured by the D4+ for the same plan.

### 2.2 Dynamic conformal arc (DCA)

As a next level of plan complexity, a dynamic conformal arc DCA was created. The arc conformed to a cylindrical‐shaped target so that the MLC shape changed from a rectangle to a circle as the arc proceeded. The base plan used a half arc from 90° to 270° and used the 6 MV energy, along with jaw tracking. This plan was modified to shift the MLC leaves by 0.25, 0.5, 1, 2, and 5 mm, disable jaw tracking and introducing gantry position errors of 1°, 2°, 5°, and 10°. Two additional plans with combined errors were also created where the MLC leaves were shifted by 1 mm and the gantry by 1° and 2°.

### 2.3 Volumetric‐modulated arc therapy (VMAT)

A baseline single‐arc VMAT plan was created using an arc from 90° to 270° with the 6MV energy and jaw tracking. This plan was modified to introduce MLC leaf shifts of 0.25, 0.5, 1, 2, and 5 mm, gantry shifts of 1°, 2°, 5°, and 10°, collimator errors of 1°, 2°, 5°, and 10°, loss of jaw tracking as well as combined errors of a 1 mm MLC leaf shift, and 1° and 2° error in gantry position. As was done for SS‐IMRT plans, all modified VMAT plans were measured with the Discover alone and a repeat delivery was performed with the D4+ phantom alone in order to directly compare Synthesis‐predicted gamma pass rates to corresponding ones from actual measurements with the D4+.

## 3 RESULTS

### 3.1 Single static field

**Figure 3:**
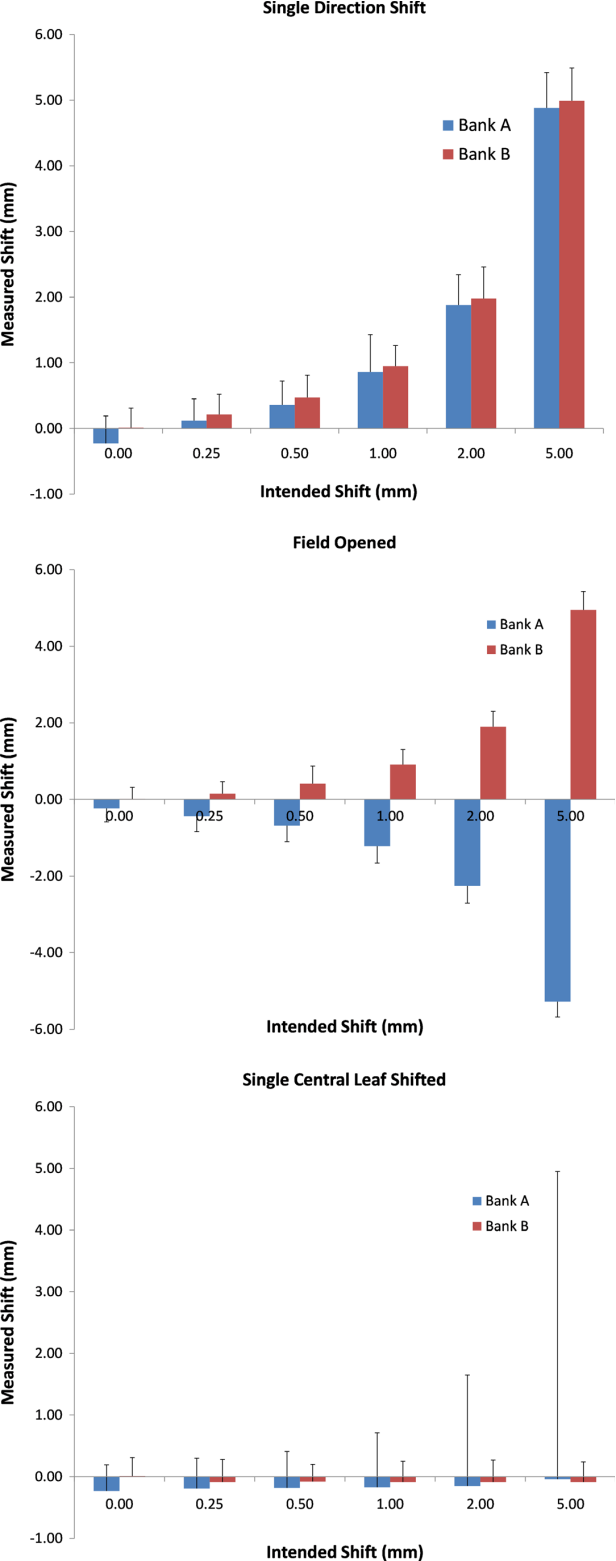
Multileaf collimator (MLC) leaf deviations for modified three‐dimensional plans. Top – leaves shifted, middle – field opened, bottom – single central leaf shifted. The average leaf deviation is reported as the height of the bars while the error bars show the maximum error reported. Note that for the single leaf error the system still detected the errors, as indicated by the much larger maximum deviation.

Figure 3 shows a plot of leaf deviations and predicted gamma pass rates for modified plans created from the baseline three‐dimensional plan (static triangular field) where the leaves were moved by known amounts. For each leaf that can be tracked, the software will calculate the leaf location based on the signal level at each diode and will then determine the deviation of that leaf compared to its planned (intended) position. A histogram of leaf deviations is then calculated for each beam, with the average and maximum deviations also presented to the user. The length of the bars represents the Discover‐reported average leaf deviation, while the error bar shows the maximum leaf deviations reported in the software for that particular field. The average detected leaf deviations were within 0.2 mm of the actual values used in the modified plans, with the maximum leaf deviation being within 0.5 mm of the intended value. Even for the case where only the central leaf was moved (bottom panel), while the *average* leaf shift was correctly identified as nearly zero, the error bars clearly show that the *maximum detected shift/error* was correctly identified (i.e., within 0.3 of the known shift that was introduced). The gamma pass rate for these plans only dropped from their baseline value for plans with a 5 mm leaf error. This was expected as the leaf position error was bigger than the distance criterion used for calculating gamma. As expected, the gamma values for the plans where only one central leaf was moved did not change compared to baseline.

**Figure 4:**
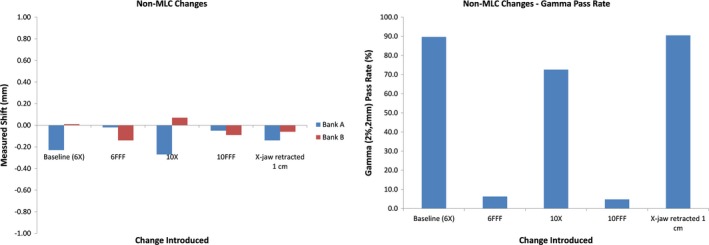
Measured leaf motion (left) and gamma pass rate (right) for plan modifications that did not include intentional leaf movement. Notice that the gamma pass rate drops significantly for instances of incorrect beam/energy. Interestingly, the 1 cm retracted × jaw apparently did not result in a significant enough change in measured fluence to trigger a noticeable drop in the gamma pass rate.

Figure 4 shows the MLC leaf deviations and predicted gamma pass rates for plan modifications to the static triangular field that did not include MLC leaf movement. As would be expected, the average measured shift in the leaves was close to 0 for all cases (left panel) as was the maximum leaf deviation reported (within 0.5 mm of intended value). The gamma pass rate (right panel) decreased significantly when the incorrect beam energy was delivered. Interestingly, the 1 cm retracted x‐jaw apparently did not result in a significant enough change in measured fluence to trigger a noticeable drop in the gamma pass rate.

### 3.2 Single IMRT field

**Figure 5:**
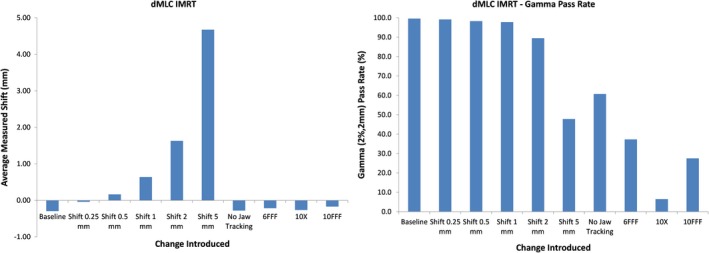
Measured leaf motion (left) and gamma pass rate (right) for modified dynamic multileaf collimator (MLC) leaf motion intensity modulated radiation therapy plans. Gamma pass rates fall dramatically once the introduced error exceeds the DTA criterion. While the detected leaf position correctly shows almost no shift when a wrong energy is used or jaw tracking is applied, the much lower predicted gamma pass rate shows the presence of an error.

**Figure 6:**
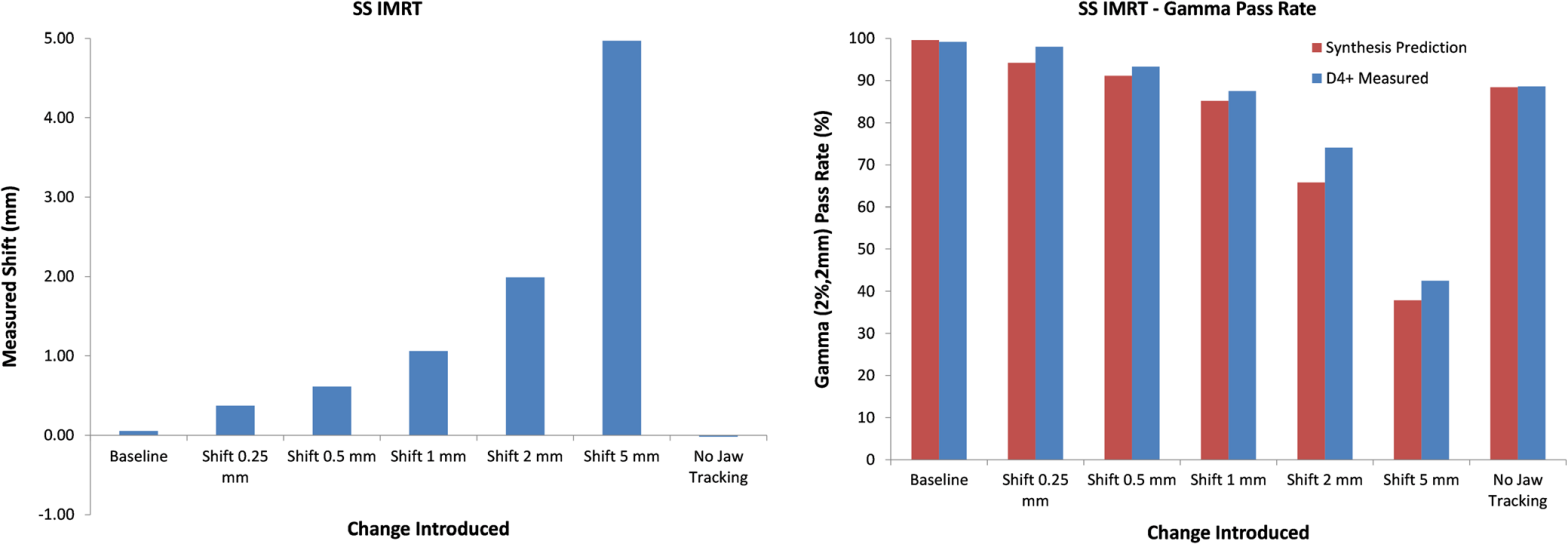
Measured leaf motion (left) and gamma pass rates (right) for modified SS plans. Also included on the right are gamma pass rates as measured with the D4+. The corresponding pattern of synthesis‐predicted gamma when compared to gamma from actual measurements confirms the accuracy of the predictions.

Figure 5 shows the average leaf deviations (left) and synthesis‐predicted gamma pass rates (right) for all modifications of the baseline dMLC plan while Fig. 6 shows the analogous results for the SS plan. The average leaf deviation is within 0.3 mm of the introduced shift but this time, the maximum leaf deviation was up to 4.5 mm different from the intended value. This difference is probably a direct result of difficulty in synchronizing the measured leaf position with the expected leaf position as further discussed in the discussion section. As expected, the predicted gamma pass rate falls dramatically once the error introduced in the leaf positions exceeds the gamma DTA criterion of 2 mm. The predicted gamma pass rate also shows a significant drop for cases where jaw tracking was disabled or where a different beam energy was used.

Figure 6 also includes comparison gamma pass rates as measured with the D4+ for the SS plans. While the average leaf deviation is within 0.3 mm of the introduced error, the maximum deviation is up to 1.5 mm different. The accuracy of the predicted gamma pass rates is confirmed by the right panel which shows that the predictions accurately mirror the behavior of the gamma pass rates from actual D4+ measurements when different changes were introduced into the baseline plan.

### 3.3 Dynamic conformal arc (DCA)

**Figure 7:**
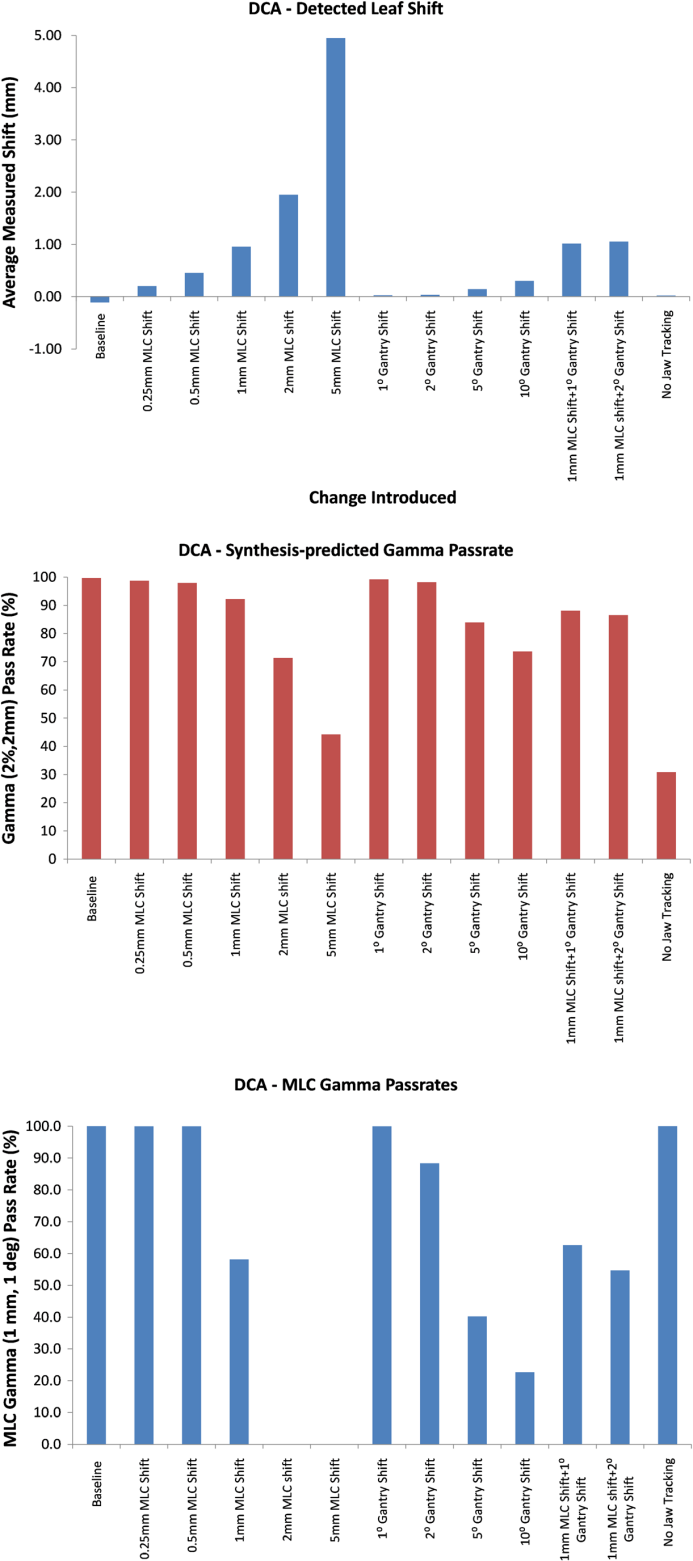
Measured leaf deviations (top), synthesis‐predicted gamma pass rate (middle), and multileaf collimator (MLC) gamma pass rates (bottom) for all variations of the dynamic conformal arc (DCA) plan evaluated. Note the drop in MLC gamma once the errors introduced exceeded the criteria of 1 mm and 1° used for this metric.

Figure 7 shows the average measured leaf deviation (top), synthesis‐predicted gamma pass rate (middle), and MLC gamma pass rate (bottom) for all plan variations of the DCA plan. Average leaf deviations were within 0.3 mm of the known errors introduced. The maximum leaf deviation was 1.7 mm for these plans. The MLC gamma is a metric available in the data analysis for all arc plans and is a criterion that is calculated by using the gamma formula and incorporating the measured difference in MLC leaf location for each detector as well as the difference in measured gantry angle instead of dose and distance‐to‐agreement. The tolerances were set to 1 mm and 1° for this test and the calculations were confirmed to work as intended, with the MLC gamma showing dramatic drops when the leaf position and gantry errors started exceeding these tolerances.

### 3.4 Volumetric‐modulated arc therapy (VMAT)

**Figure 8:**
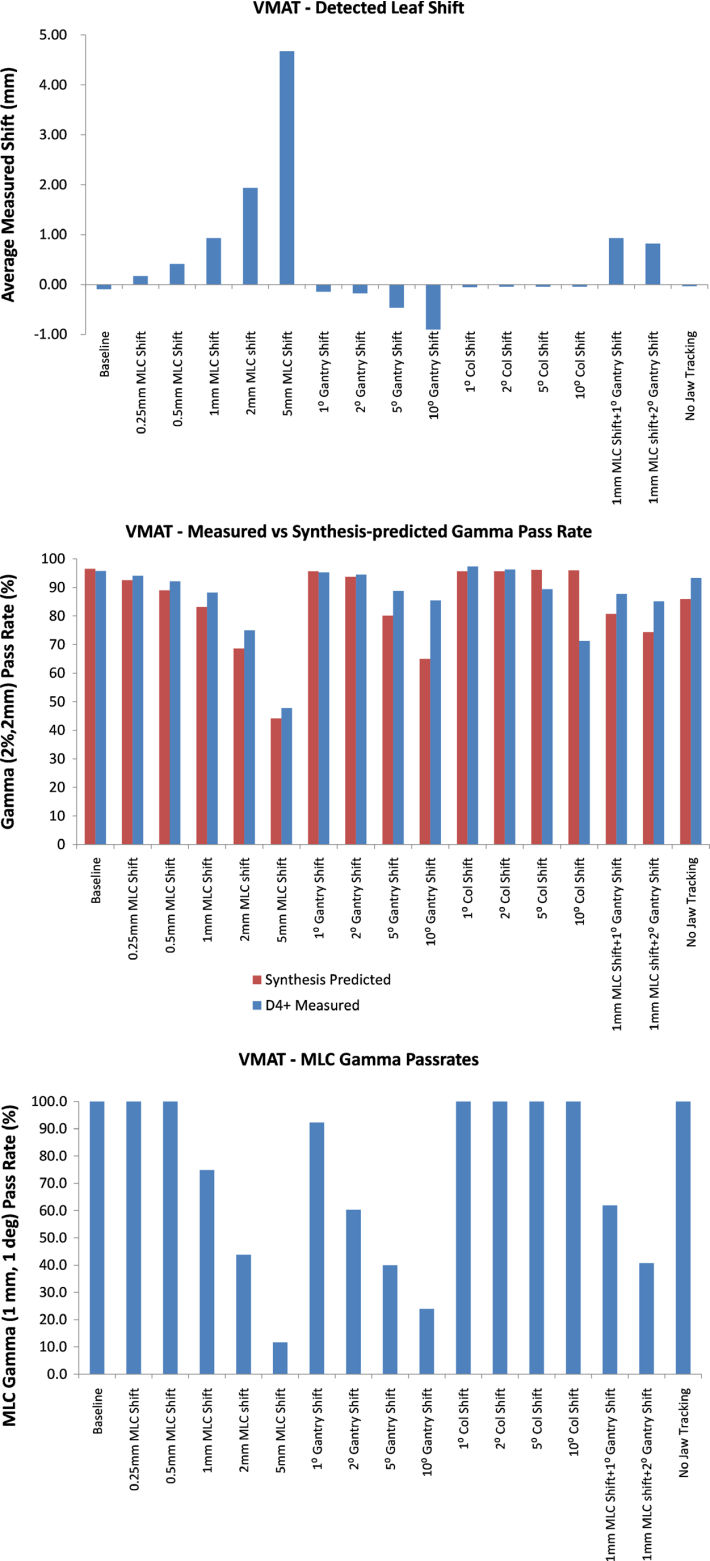
Measured leaf deviations (top), synthesis‐predicted and D4+‐measured gamma pass rate (middle), and multileaf collimator (MLC) gamma pass rates (bottom) for all variations of the dynamic conformal arc (DCA) plan evaluated. While the D4+‐measured gammas generally agree with the synthesis‐predicted gammas (middle panel), the one exception is for changes in collimator position which is not identified by the synthesis predictions.

Figure 8 shows the average measured leaf deviation (top), synthesis‐predicted and D4+‐measured gamma pass rate (middle), and MLC gamma pass rate (bottom) for all plan variations of the VMAT plan. Average detected leaf shifts agree with the known value introduced within 0.8 mm. However, the maximum differences for individual leaves were on the order of 25 mm, much higher than seen for DCA fields. Since the VMAT fields are basically analogous to a dMLC delivery, with the beam being constantly on while both the gantry and MLC leaves are moving, the synchronization of planned and measured leaf position is now the most complex encountered, possibly also explaining why the mean difference now within 0.8 mm compared to the 0.3 mm level seen for other types of plans. Once again, the synthesis‐predicted gammas are very close those from actual D4+ measurements. We note, however, that the predictions fail to show changes in collimator positions. This is expected behavior because the MLC gamma does not consider errors of collimator position. The MLC gamma metric shows the expected behavior when the changes made in the baseline plan exceed the 1 mm, 1° criteria used for the metric.

## 4 DISCUSSION

The results shown in Fig. 3 confirm that the Discover unit can detect submillimeter changes in leaf positions. Of note was the fact that even a single leaf being incorrectly positioned was correctly identified by the system. For static beams, with the maximum detected leaf position deviation being within 0.5 mm of the actual error introduced, the results confirm that this device can fulfill the TG‐142^36^ requirement of detecting leaf position repeatability to within 1 mm. As for IMRT plans, the report requires 95% of error counts to be <3.5 mm. While the software does offer statistics on average and maximum leaf deviations, the current implementation of the software does not directly provide this information, the distribution of leaf deviations is provided so that this information can be deduced.

Figure 4 shows the importance of using the Discover in synthesis mode rather than as express measure only. As previously explained, the Discover unit cannot convert the measured fluence to dose without initially being synchronized with measurements from the Delta4+phantom. Therefore, while the device correctly measured that the leaves were correctly positioned when a different energy was used, there would be no way for it to detect that the fluence measured does not correlate with the dose distribution expected. In that case, the field would pass QA and no flag would be raised. However, when initially synchronized in Synthesis Mode, the Discover device can then correctly interpret that the fluence delivered was not correct for subsequent measurements using the device by itself.

For IMRT measurements, the average leaf deviation measured was within 0.3 mm of the deviations introduced into the plan for both dMLC and SS plans. The maximum detected deviation (error bar length) for dMLC plans is roughly 5 mm while the noise for SS plans is around 1.5 mm. Although the SS leaf deviation measurements would catch the maximum leaf error of 3.5 mm as recommended by TG142, this would not necessarily be the case for all leaves for the dMLC plans. Since the Discover and D4+ measures every pulse of radiation emitted by the linac, the dramatic increase in uncertainty of leaf position detection for dMLC plans is likely due to synchronization mistakes in measuring the location of leaves for each control point to compare to planned locations. As opposed to an SS field, where the shape is fully created prior to dose being delivered, the dMLC plan is much harder to synchronize with because, at least on the Truebeam platform, the leaves are constantly moving while the dose is being delivered. Even small synchronization mistakes will likely make the measured leaf position very different from the planned leaf position.

Once again, the importance of synthesis mode is obvious for the dMLC plans where different energies were used or when jaw tracking was disabled. With the leaves going to the correct position for these measurements, an Express Measure measurement would not raise any flags. However, synthesis‐predicted gamma pass rates definitely point to an error occurring during the delivery because the widely different fluence measured is “synthesized” into a D4+ dose distribution, which would be very different from the baseline. It is also interesting to see that D4+ measured dose distributions had gamma pass rates that followed the same trend as seen with the synthesis‐predicted pass rates, although they tended to always be higher, meaning the synthesis‐predicted gamma values would tend to point to a possible error in a delivery earlier than the actual measurement would.

For arc plans, the average leaf deviations were very close to the actual deviations introduced in the plans, both for DCA and VMAT plans. Once again, the maximum detected leaf deviation was much larger for VMAT plans, on the order of 10 mm compared to the values of 2 mm for DCA plans. This is also likely due to difficulties in synchronizing measured leaf positions with planned leaf positions for VMAT plans where large changes are occurring in leaf position with gantry angle, as opposed to DCA plans where the position the MLC leaves changed very little with gantry position.

The maximum detected deviations were much higher than expected when gantry angle deviations were introduced. For instance, these deviations went as high as 6 mm when a 10° gantry shift was included for DCA plans. The corresponding value was 25 mm for VMAT plans. These large leaf position errors are attributable to leaf pairs that are only part of the field for a short time and then move out of the field, under the secondary jaws. Due to the synchronization challenges, these large motions are captured as leaf position errors.

For VMAT plans, the gamma pass rates for dose distributions measured with the D4+ generally followed the same trend as synthesis‐predicted values, with the actual dose measurements having pass rates that were generally higher, just as was observed for SS plans. The one big difference was for plans with collimator changes. In general, the predicted gamma pass rates stayed almost constant, even when collimator angles were changed by as much as 10°. This was clearly detected as a delivery error when the doses were measured with the D4+. This behavior is due to the fact that the Discover rotates with the collimator such that any collimator rotation results in no change to the fluence measured. The Discover, however, does have a gyroscope that will measure the collimator angle. For the plan variations tested here, the collimator location was correctly measured within 1° and the measurement software allows the user to set a pass/fail criterion for the field that includes any observed collimator deviation.

## 5 CONCLUSION

For the Discover system to detect all errors simulated here, including plans delivered using the wrong energy, or failure of the jaws to be positioned correctly, the Discover should be used in Synthesis Mode. When used this way, average leaf motion differences detected by the Discover system were within 0.5 mm of the known introduced error for all plan types and MLC gamma value distributions showed high consistency between errors introduced in the plan and the criteria used for the gamma calculations. As such, the Discover device promises to be an effective, near real‐time detection system for capturing potentially disastrous dose delivery errors. However, for those clinics that do not possess the D4+ as well as the Discover, the device can still be used in “Express Measure” mode and the device can still be used to verify the correct position of the MLC leaves and gantry and collimator for each beam.

## CONFLICT OF INTEREST

Drs. Sarkar, Szegedi, and Salter have been reimbursed for meeting registration and travel costs by Scandidos Inc.
